# Identification and characterization of miRNAs and *PHAS* loci related to the early development of the embryo and endosperm in *Fragaria* × *ananassa*

**DOI:** 10.1186/s12864-022-08864-3

**Published:** 2022-09-08

**Authors:** Xiaotong Jing, Hong Zhang, Xinjia Huai, Qi An, Yushan Qiao

**Affiliations:** Laboratory of Fruit Crop Biotechnology, College of Horticulture, Nanjing Agricultural University, No. 1 Weigang, Nanjing, Jiangsu 210095 People’s Republic of China

**Keywords:** MicroRNA, *PHAS* loci, Strawberry, Embryo and endosperm development

## Abstract

**Background:**

The strawberry fleshy fruit is actually enlarged receptacle tissue, and the successful development of the embryo and endosperm is essential for receptacle fruit set. MicroRNAs (miRNAs) and phased small interfering RNAs (phasiRNAs) play indispensable regulatory roles in plant growth and development. However, miRNAs and phasiRNAs participating in the regulation of strawberry embryo and endosperm development have yet to be explored.

**Results:**

Here, we performed genome-wide identification of miRNA and phasiRNA-producing loci (*PHAS*) in strawberry seeds with a focus on those involved in the development of the early embryo and endosperm. We found that embryos and endosperm have different levels of small RNAs. After bioinformatics analysis, the results showed that a total of 404 miRNAs (352 known and 52 novel) and 156 *PHAS* genes (81 21-nt and 75 24-nt genes) could be found in strawberry seed-related tissues, of which four and nine conserved miRNA families displayed conserved expression in the endosperm and embryo, respectively. Based on refined putative annotation of *PHAS* loci, some auxin signal-related genes, such as *CM3*, *TAR2*, *AFB2*, *ASA1*, *NAC* and *TAS3,* were found, which demonstrates that IAA biosynthesis is important for endosperm and embryo development during early fruit growth. Additionally, some auxin signal-related conserved (miR390-*TAS3*) and novel (miR156-*ASA1*) trigger-*PHAS* pairs were identified.

**Conclusions:**

Taken together, these results expand our understanding of sRNAs in strawberry embryo and endosperm development and provide a genomic resource for early-stage fruit development.

**Supplementary Information:**

The online version contains supplementary material available at 10.1186/s12864-022-08864-3.

## Background

Cultivated strawberry (*Fragaria* × *ananassa* Duch.) is one of most popular berry crops in the Rosaceae family, and China has been the world’s largest strawberry producer since 1994 [1]. In the botanical sense, the strawberry fruit is neither a berry nor a true fruit; it is actually a group of achenes dotting the receptacle’s surface. Total or partial removal of the achenes from the receptacle resulted in failure of the fleshy fruit to grow and ripen or an abnormal shape unless exogenous auxin was supplied [2, 3]. Achenes are rich sources of auxin, whose concentration peaks during early development and then declines quickly as fruit growth and ripening progress. This is consistent with the levels of the auxin-related amino acid tryptophan (Trp) and the lack of free auxin in the receptacles during all developmental stages [2, 4–6]. This auxin level pattern correlates with auxin promoting receptacle enlargement and inhibiting strawberry ripening [7, 8]. In contrast to the auxin level, the abundance of abscisic acid (ABA) in achenes remained constant during early development and then increased at the onset of fruit ripening [9]. Moreover, ABA is self-induced in achenes and coordinates with IAA in the regulation of early fruit development and later ripening in strawberry [9]. As a consequence, achenes are essential to strawberry receptacle enlargement and ripening. Each achene consists of four tissues: a hard and relatively thick ovary wall, a thin seed coat, a single-layer endosperm, and a small embryo [6, 10]. Among them, the endosperm is a nourishing tissue that supports embryo growth and germination, and failure of endosperm development will cause embryo arrest [11]. In addition, endosperm may play a more prominent role than the embryo in the biosynthesis of auxin and gibberellin for strawberry fruit set [10]. In this regard, the role of endosperm is widely accepted as being altruistic. However, the embryo also regulates the development of the endosperm; for example, scanning electron microscopy (SEM) photos that show the decomposition of starch granules in the endosperm cells facing the scutellum of the embryo [12], and *VP1* genes expressed in or GA/ABA signalling released from the embryo modulate endosperm development [13–15]. Therefore, successful communication between the endosperm and embryo is necessary for seed development and has a profound impact on fruit set and quality.

At present, many studies have revealed that small RNAs (sRNAs), such as miRNAs and short interfering RNAs (siRNAs), are key genetic and epigenetic regulators in diverse biological processes related to plant growth, development, and stress responses [16]. miRNAs are the most abundant sRNAs and show wide conservation in plants. Mature miRNAs are 20–22 nucleotides in length, are derived from single-stranded RNAs, and regulate their target genes through either transcript cleavage or translation inhibition [17]. Distinct from miRNA biogenesis, siRNAs are generated from long double-stranded RNAs, and distinct siRNA species from both strands depend on the Dicer proteins involved. Accordingly, siRNAs can be further grouped into several subclasses, including heterochromatic small interfering RNAs (hc-siRNAs), phasiRNA and other endogenous siRNAs [18]. Among them, hc-siRNAs are predominantly 24 nt long and are mainly derived from repetitive sequences or transposons. They direct de novo cytosine DNA methylation at homologous loci through an RNA-directed DNA methylation (RdDM) pathway [19]. PhasiRNAs are a special type of siRNA that rely on the function of miRNAs in their biogenesis. After cleavage of the target miRNA, the 5’ fragment of the target mRNA is rapidly degraded and the 3’ fragment is converted into dsRNA via the activity of RNA-DEPENDENT RNA POLYMERASE6 (*RDR6*). Then, the dsRNA is iteratively cleaved by a Dicer protein (DCL4 or DCL5) from the strand containing the cleavage site, producing duplexes of phasiRNAs (DCL4 forms 21-nt phasiRNAs, DCL5 forms 24-nt phasiRNAs). The biogenesis of phasiRNA is guided by miRNAs through either one or two miRNA binding sites [17]. PhasiRNA-producing loci are named *PHAS* loci, and the first *PHAS* loci, *TAS3,* was identified in *Arabidopsis* as a conserved noncoding genes among land plants. The *TAS3* gene targeted by miR390 generates several tasiRNAs that target AUXIN RESPONSIVE FACTOR (*ARF*) genes [20]. The three components, miR390, *TAS3* and *ARF*, are indispensable regulators in auxin signalling, and the miR390-*TAS3*-*ARF* pathway plays an important role in leaf morphogenesis and flower development [21, 22]. PhasiRNAs are also generated from protein-coding genes; for instance, the PENTATRICOPEPTIDE REPEAT (*PPR*), nucleotide-binding leucine-rich repeat receptor (*NB*-*LRR*) and v-myb avian myeloblastosis viral oncogene homologue (*MYB*) families were shown to generate phasiRNAs in *Arabidopsis* [23], *Medicago* [24], apple (*Malus* × *domestica* ‘Golden delicious’) [25], litchi (*Litchi chinensis* ‘Huaizhi’) [26], woodland strawberry (*Fragaria vesca*) [27] and *Rosa rugosa* [28].

To date, research on sRNAs in strawberry has mainly focused on fruit ripening [29, 30] and stress [31], but there is little research on seed (embryo and endosperm) development. In this study, we focused on the developing embryo and endosperm at the early development stage (12 days after pollination (DAP)) by genome-wide characterization of miRNAs, their target genes and the *PHAS* pathway. Our results demonstrate the large miRNA population and some miRNA-*PHAS* pairs in the embryo and endosperm, which enhances our understanding of plant sRNAs in strawberry seed development.

## Results

### Library construction and sequencing

**Fig. 1:**
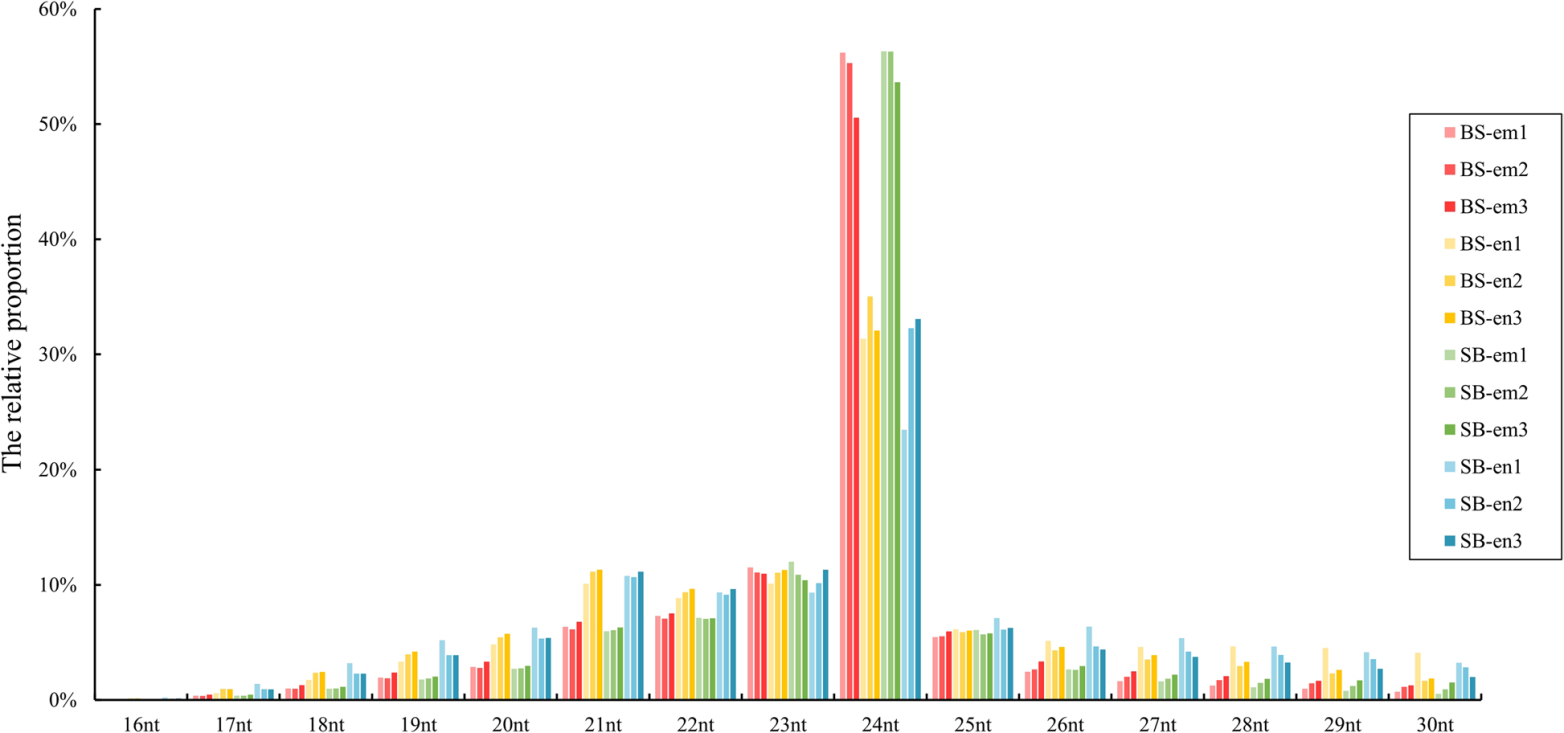
Length distribution and abundance of sRNAs in the reciprocally crossed embryo and endosperm of strawberry. BS and SB represent ‘Benihoppe’ (♀) × ‘Sweet Charlie’ (♂) and ‘Sweet Charlie’ (♀) × ‘Benihoppe’ (♂), respectively. em: embryo; en: endosperm. For each tissue, three biological replicates (em1, em2, and em3; en1, en2, and en3) were performed

To identify known and novel miRNAs in the embryo and endosperm of strawberry, twelve sRNA libraries derived from reciprocally crossed embryo and endosperm tissues of *F.* × *ananassa* cultivar ‘Benihoppe’ and ‘Sweet Charlie’ at 12 DAP were constructed and sequenced. Initially, a total of 9,935,499 to 54,785,143 raw reads were obtained for each library (Table S1). After filtering the low-quality reads, adaptor and contaminant sequences, reads with lengths < 16 nt and reads containing polyA in the small RNA fragment, 8,100,514 to 44,609,527 clean reads were obtained for each library (Table S1). We analysed the length distribution of the sequences in the 12 libraries and found that the majority of sRNAs ranged from 20 to 25 nt in length. The concentrated length distribution peaked at 24-nt sRNAs, while 23-nt and 22-nt sRNAs were the second and third most abundant populations in each library, respectively. Furthermore, there were more 24-nt sRNAs present in the embryo than in the endosperm, whereas the 21–22-nt sRNA showed a reversed trend in the embryo and endosperm of BS, which may be due to tissue-specific expression of sRNA in strawberry development (Fig. 1). Moreover, all clean reads were aligned against the GenBank database [32], Rfam 11.0 database [33], and cultivated strawberry genome [34]. An average of 37.16%, 0.76%, 0.14%, and 2.58% reads matched other noncoding RNAs, including rRNAs, tRNAs, snRNAs, and snoRNAs, and these sRNA populations contained more reads in the endosperm than in the embryo at the 12 DAP stage. In addition, a small portion of reads was mapped to protein coding sequences and intron sequences, which are likely to be RNA degradation products (Figure S1).

### Identification of known and novel miRNAs in the developing embryo and endosperm

**Fig. 2:**
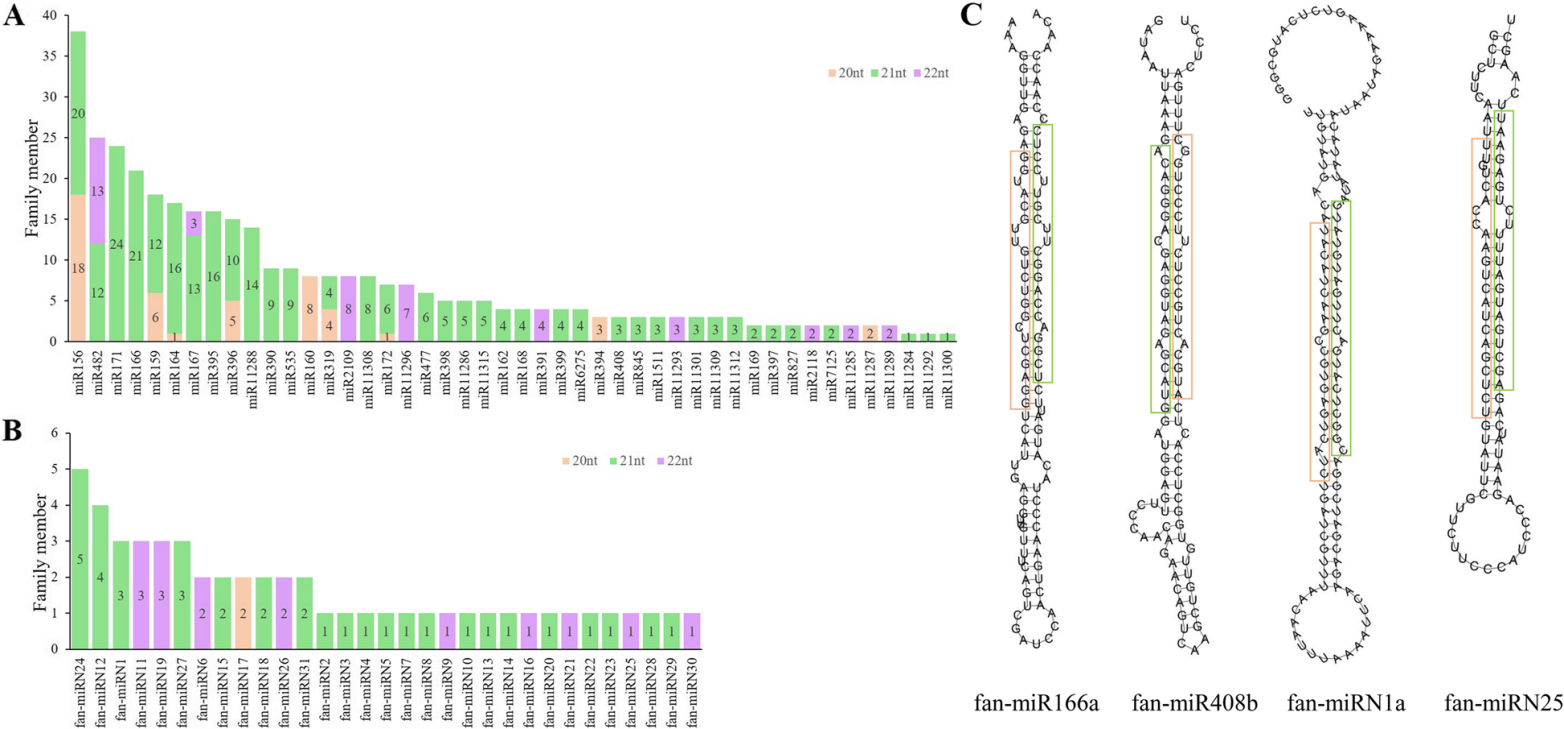
The number of members in known and novel miRNA families. **A** A total of 46 known miRNA families were identified in developing strawberry embryos and endosperm. **B** Fifty-two novel miRNAs belonging to 30 novel miRNA families were identified in embryos and endosperm. Different colours are used to present the length of each miRNA family member.  denotes 20 nt miRNA,  21 nt miRNA and  22 nt miRNA. **C** The secondary structures of fan-miR166a, fan-miR408b, fan-miRN1a, and fan-miRN25 identified from strawberry. Mature miRNA and predicted miRNA* sequences are marked in  and  , respectively

Clean reads (excluding reads mapped to snRNAs, snoRNAs, tRNAs and coding sequences or intron sequences) from 12 libraries were aligned against the miRbase 22 database [35]. A total of 352 known miRNAs were identified (Table S2); these miRNAs belong to 46 miRNA families. Twenty-two out of the 46 families are highly conserved in plants, and a few less conserved miRNAs, including miR477, miR482/2118, and miR535, were identified. As shown Fig. 2A, the most abundant miRNA families are miR156 (38 members), miR482 (25 members), and miR171 (24 members). In addition, 52 novel mature potential candidate miRNAs from 12 libraries were identified, of which most were 21 nt in length (Fig. 2B & Table S2). Pri-miRNAs with stem‒loop structures are cleaved into miRNA::miRNA* duplexes, and in this study, we also analysed the miRNA* structure (Fig. 2C & Table S2). The abundance of miRNA* was significantly lower than that of mature miRNAs; in contrast, we found that fan-miR408* and fan-miRN1* were more abundant than their references, fan-miR408 and fan-miRN1. Interestingly, miR408* was also found to have a higher abundance than rco-miR408 in caster been [36].

### Expression profiling of miRNAs in the developing embryo and endosperm

**Fig. 3:**
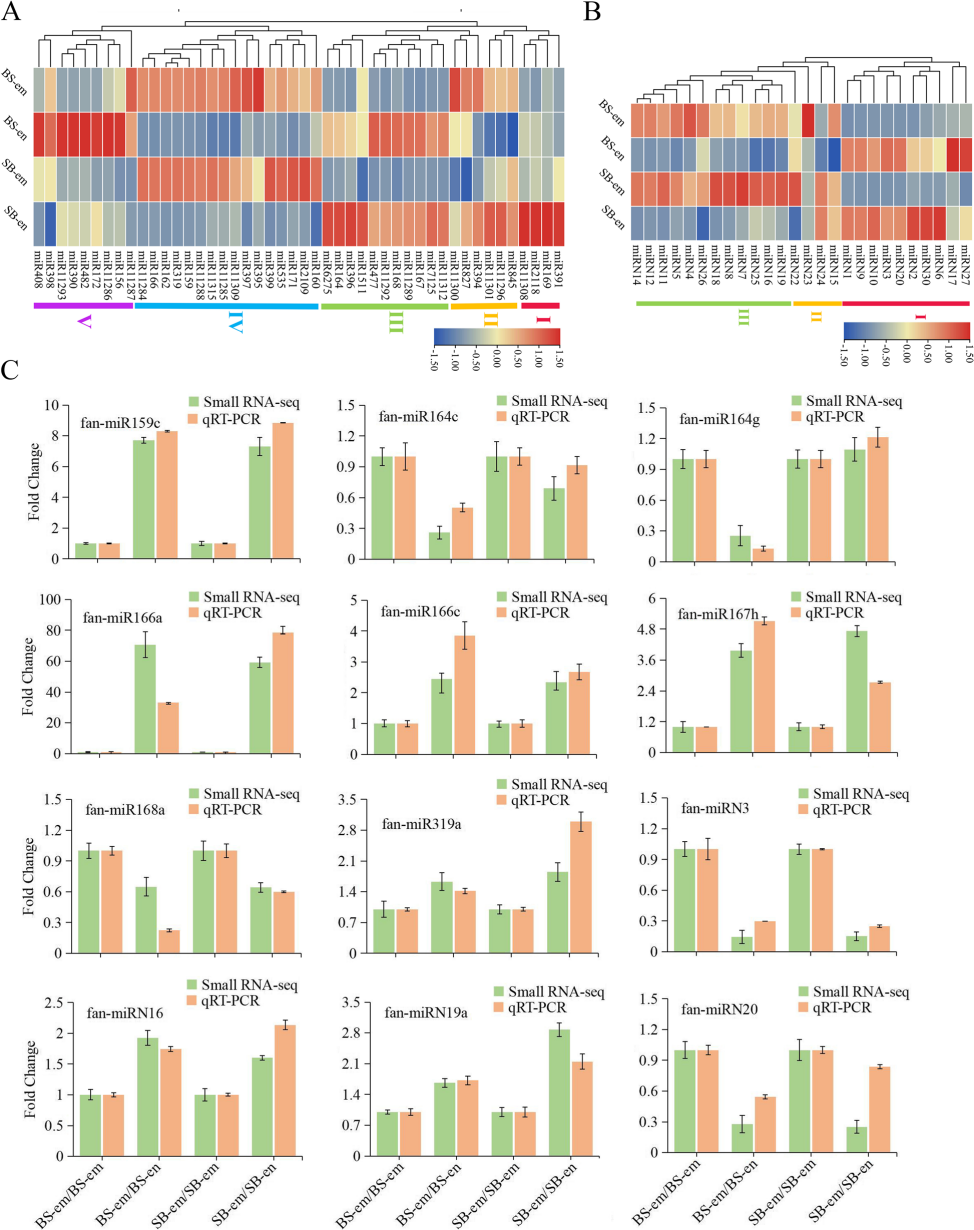
Expression profiling of known and novel miRNA families in the developing strawberry embryo and endosperm. **A** Forty-six known miRNA families were divided into five groups according to their expression patterns in the embryo and endosperm. **B** Twenty-six novel miRNA families were classified into three groups. **C** Tailing-reaction qRT‒PCR verification of 12 differentially expressed miRNAs in the development of strawberry embryos and endosperm. The error bar represents the error values of three biological replicates

To explore known miRNA families that are highly expressed in the development of embryos and endosperm in strawberry, the TPM reads of miRNA families were counted by summing. Among them, miR1511 was the most abundant family, followed by miR156 and miR159 (Figure S2). With these expression data, we can examine how highly expressed miRNA families are involved in the embryo and endosperm. These high expression levels of known highly expressed miRNA families were divided into five groups according to their expression profile (Fig. 3A). Among them, group I and group V were highly expressed in the endosperm of SB and BS, respectively, and we considered that this was related to variety specificity. The expression levels of group III miRNA families, including miR164, miR167, miR168, miR396, miR477, miR1511, miR6275, miR7125, miR11289, miR11292 and miR11312, decreased in the embryo but increased in the endosperm, indicating that their functions are potentially involved in the development of the endosperm. In contrast, group IV had a high expression level in the embryo from the reciprocal cross, which suggested that these miRNA families play an important role in the embryo. Novel miRNA families with more than 50 TPM reads were found to be highly expressed in the development of strawberry seeds. Of them, 26 novel miRNA families were classified, and miRN12 was the most abundant family, followed by miRN10 and miRN8. According to the different expression levels between the embryo and endosperm, these novel families were divided into three groups (Fig. 3B). Ten miRNA families showed upregulated expression in the endosperm from the reciprocal cross relative to the embryo, so we placed these in group I. In group II, miRN23 showed tissue-specific expression in the embryo of BS, and miRN24 showed strain-specific expression in SB. Group III miRNA families have upregulated expression in the embryo from reciprocal crosses, showing their regulatory function in heart embryo development. To verify the results of sRNA sequencing, tailing-reaction quantitative reverse transcription PCR (qRT‒PCR) was performed to determine the expression level of 12 differentially expressed miRNAs in the development of strawberry embryos and endosperm. As shown in Fig. 3C, the expression patterns of miRNAs determined by qRT‒PCR were broadly consistent with those from sRNA sequencing data.

### Prediction and analysis of miRNA target genes in the embryo and endosperm

**Fig. 4:**
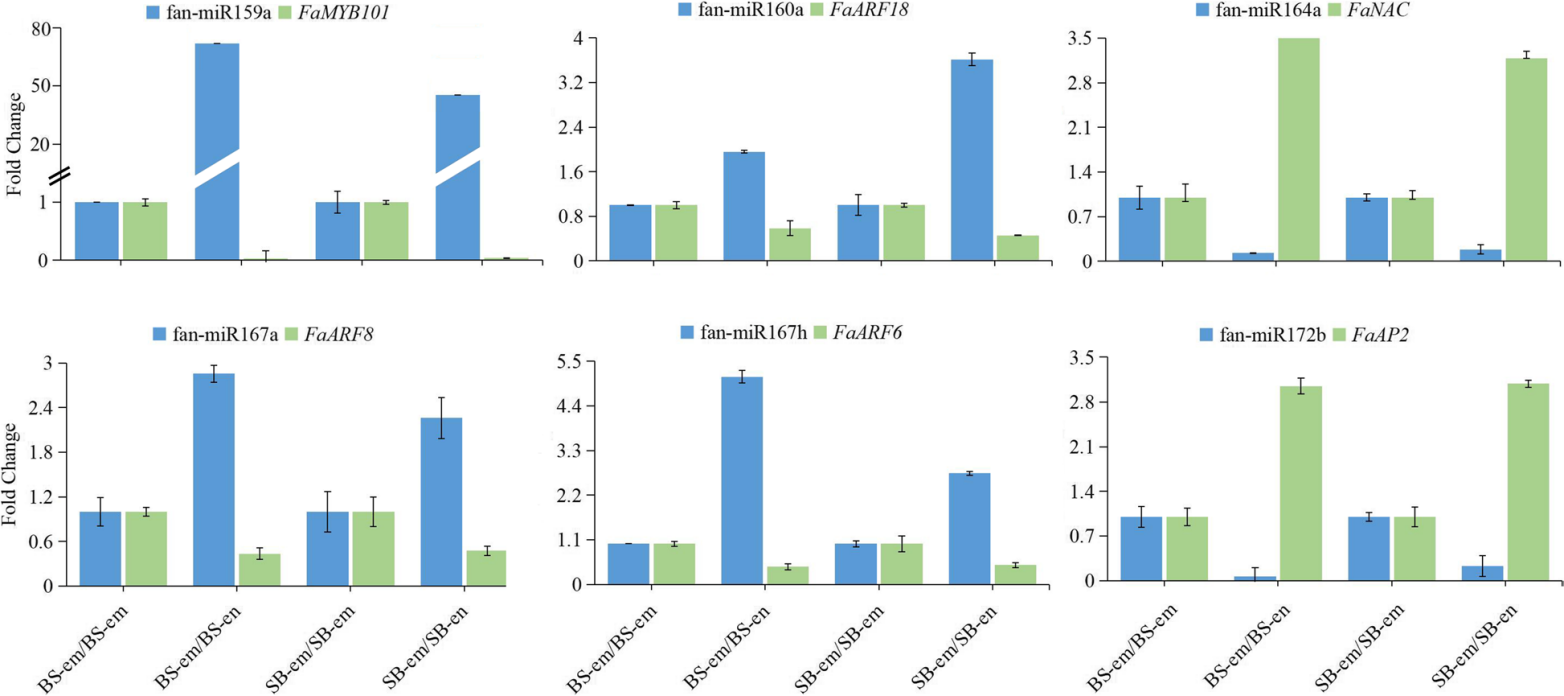
Quantitative real-time PCR validation of six identified miRNA‒mRNA pairs in strawberry. Blue and green bars represent the miRNAs and their corresponding target mRNAs, respectively. The error bar represents the error values of three biological replicates

To analyse the function of miRNAs in strawberry embryo and endosperm development, we predicted targets of the 404 miRNA candidates, including 352 known miRNAs and 52 novel miRNAs; 2984 target genes were detected (Table S3). The majority of conserved miRNA targets are various transcription factors, including *SPL*, *MYB*, *ARF*, *NFYA*, *AP2*, *TCP2*, and *HD*-*ZIP*, which play an important role in regulating plant development [16, 37]. Other conserved miRNA targets include *NAC* (miR164), scarecrow-like protein (miR171), *TAS3* (miR390), sulfate transporter (miR395), laccase genes (miR397), umecyanin-like (miR398), ubiquitin-conjugating enzyme or inorganic phosphate transporter (miR399), uclacyanin (miR408) and SPX domain-containing membrane protein/protein TORNADO 2 (miR827). miR162 and miR168 were found to target genes encoding DCL1 and AGO1, respectively, which are key components of the general process of miRNA biogenesis and activity. miR391, miR482, miR2109, miR2118 and three novel miRNA families containing miRN19, miRN25 and miRN29 were found to target disease resistance proteins. F-box genes are a large family in plants; approximately 800 of these genes in *F. vesca* are involved in different biological events [16, 38] and have been identified to be targeted by the conserved miRNA of miR394, the less conserved miRNAs including miR11288 and miR11315, and two novel miRNA families, miRN1 and miRN20. Two less conserved (miR11285 and miR11301) and two novel (miRN24 and miRN26) miRNA families targeted the PPR protein. Receptor-like serine/threonine protein kinases are a large family of biological enzymes that participate in the phosphorylation of proteins [39]; these kinases were targeted by several miRNA families, including miR390, miR482, miR535, miR7125, miR11284 and miR11308. Moreover, novel miRNAs targeted a number of functional genes, such as NEDD8-specific protease 1 (miRN3), RNA polymerase II degradation factor 1-like (miRN4), sulfate transporter (miRN5), elongation of fatty acids protein (miRN11), ACT domain-containing protein (miRN14), piriformospora indica-insensitive protein (miRN17), protection of telomeres protein (miRN18), and phosphoenolpyruvate carboxylase (miRN31). To further demonstrate that these identified genes were targeted by miRNA, qRT‒PCR was performed to analyse the fan-miR159a-*FaMYB101*, fan-miR160a-*FaARF18*, fan-miR164a-*FaNAC*, fan-miR167a-*FaARF8*, fan-miR167h-*FaARF6*, and fan-miR172b-*FaAP2* pairs. The qRT‒PCR results showed that these 6 target gene expression profiles were opposite those of their corresponding miRNAs (Fig. 4), which was consistent with previous studies; for instance, miR167 directly targets *ARF6* and *ARF8* and decreases their transcript levels, which is essential for the fertility of both ovules and anthers [40]. miR172 inhibits the expression of *AP2*, which limits cell division and expansion in *Arabidopsis* [41], and overaccumulation of miR172 leads to the silencing of *AP2* in apple [42].

### Identification of *PHAS* loci in the developing embryo and endosperm

To investigate phasiRNA biogenesis and regulation in embryos and endosperm, *PHAS* loci were identified based on small RNA reads by using sRNAanno [43]. Hundreds of *PHAS* loci (251 twenty-one-nucleotide *PHAS* loci and 798 twenty-four-nucleotide *PHAS* loci) were identified. When using a stringent phase score (phase score >  = 10), 81 21-nt *PHAS* loci could still be identified. We identified 75 24-nt *PHAS* loci by using a phase score of 5 (phase score >  = 5) (Table S4).

**Fig. 5:**
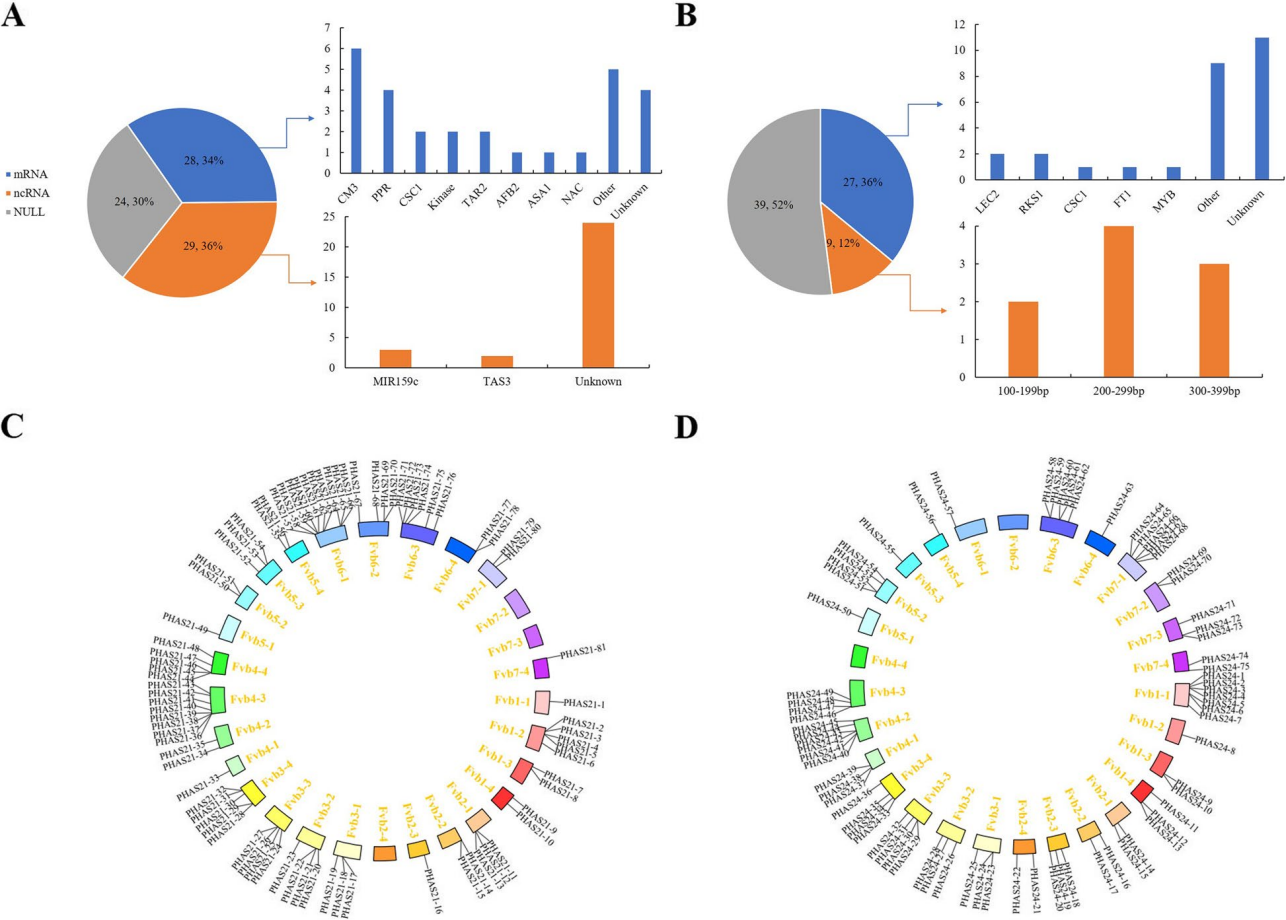
Classification of identified *PHAS* loci. **A** The 21-nt *PHAS* loci were classified into three categories according to their functional annotation. *CM3*, chorismate mutase 3; *PPR*, pentatricopeptide repeat; *TAR2*, tryptophan aminotransferase-related 2; *AFB2*, auxin signalling F-box 2; *ASA1*, anthranilate synthase alpha subunit 1; *NAC*, NAM, ATAF and CUC proteins. **B** Classification of 24 identified *PHAS* loci. Coding (blue) genes were classified according to their annotation. *LEC2*, B3 domain-containing transcription factor *LEC2*; *RKS1*, G-type lectin S-receptor-like serine/threonine-protein kinase *RKS1*; *FT1*, flowering locus T1. Noncoding (orange) loci are grouped based on the length of the *PHAS* loci. **C** 21-nt *PHAS* loci located in the strawberry genome. **D** 24-nt *PHAS* loci located in the strawberry genome

To refine the putative annotation of the predicted *PHAS* loci, we aligned these *PHAS* loci to the NCBI Nucleotide Collection (nr/nt) database and the PlantRep database (Table S4). Among the 21-nt *PHAS* loci, some *PHAS* loci were protein coding genes, such as *PPR*, *TAR2*, *AFB2*, *NAC* and kinase, which are conserved in plants [44, 45] (Fig. 5A). We found that six *CM3* genes and one *ASA1* gene in strawberry also generated 21-nt phasiRNAs, which play an important role in the shikimic acid pathway [46]. In addition, 36% of the *PHAS* loci originated from ncRNA, and two identified 21-nt *PHAS* loci were *TAS3* loci; we did not find *TAS1* and *TAS2* loci in strawberry. The 81 *PHAS* loci were mapped to the genome using TBtools [47] software, and among them, the majority of *PHAS* loci were clustered on chromosomes Fvb4-3 and Fvb6-1 (Fig. 5C). Up to 55 *PHAS* loci overlapped with annotated protein-coding genes of cultured strawberry, including *CM1*/*3*, *PPR*, peptidyl-prolyl cis–trans isomerase *FKBP62*, *TAR2*, DNA repair protein, E3 ubiquitin-protein ligase, *AFB2*, and other proteins (Table S4). A large number (33/55) of *PHAS* loci overlapped with genes of unknown function, many of which were single copies in the genome, suggesting cis- rather than trans-activity. The remaining 26 *PHAS* loci were found in intergenic regions of the genome (Table S4).

Many 24-nt PHAS loci originated from protein coding genes, such as *LEC2*, *RKS1*, *CSC1*, *FT1* and *MYB* (Table S4). In addition to protein coding genes, 9 predicted noncoding genes produced abundant phasiRNAs, which were of short length (100 bp ~ 400 bp) based on sRNA data (Fig. 5B). Interestingly, we found that most 24-nt *PHAS* loci (61.33%) were located in the repeating sequence region, which is different from the 21-nt *PHAS* loci (Table S4). The majority of *PHAS* loci were clustered on chromosomes Fvb1-1, Fvb4-2, Fvb6-3 and Fvb7-1 (Fig. 5D). Seventy-two percent (54/75) of the *PHAS* loci were located in intergenic regions of the genome, and 28% (21/75) of the *PHAS* loci overlapped with annotated protein-coding genes in cultured strawberry. Interestingly, these annotated protein-coding genes are enzymes, except for uncharacterized proteins, including DNA ligase, isoleucine–tRNA ligase, RNA helicase, protein phosphatase 2C, methylesterase 17, xyloglucan galactosyltransferase KATAMARI1 and key players in epigenetic modification, including the ENHANCED DOWNY MILDEW 2 (EDM2) protein, the helicase protein MOM1, and the methyltransferase PMT9 (Table S4).

### The expression patterns of *PHAS* loci in the developing embryo and endosperm

**Fig. 6:**
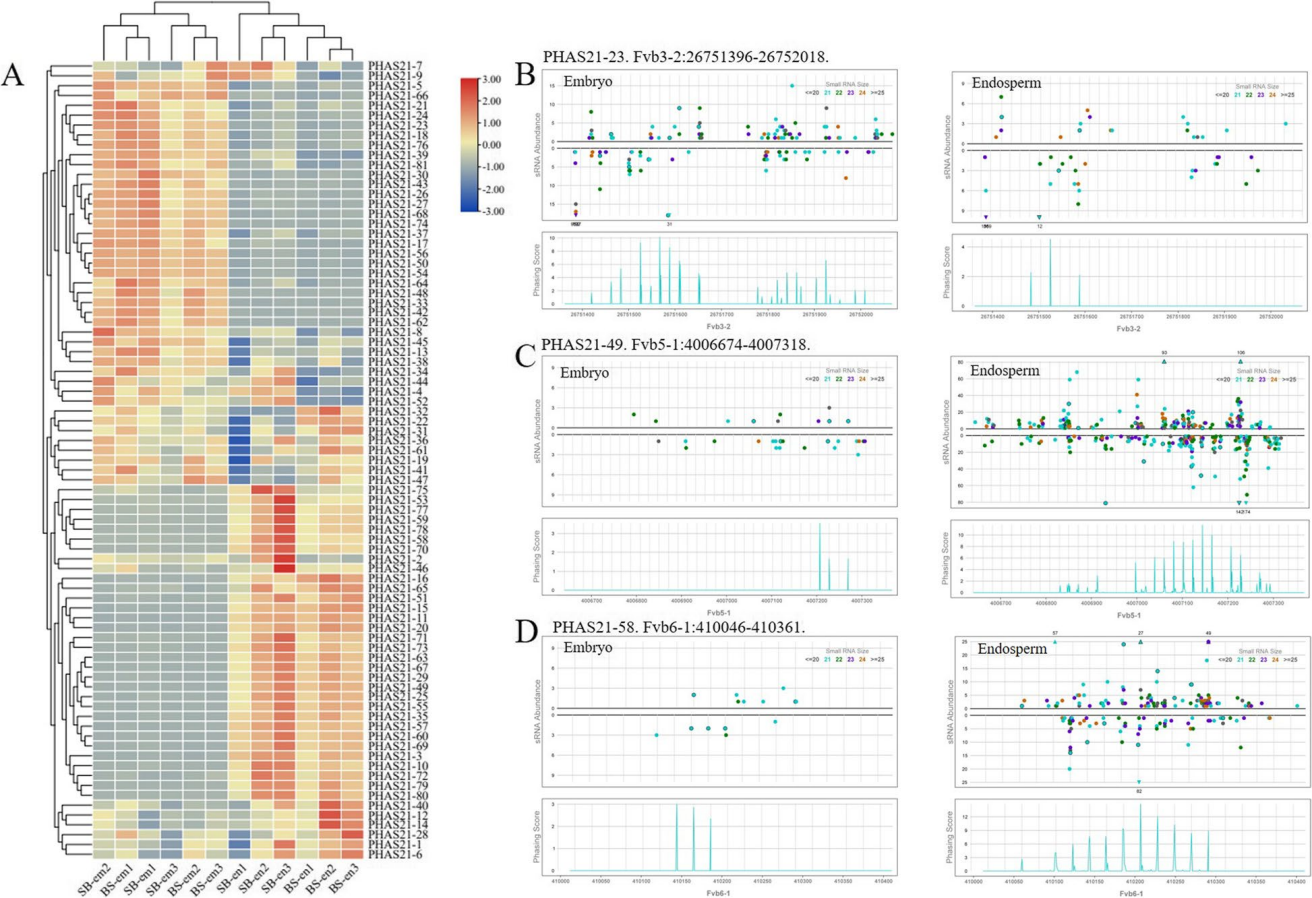
Expression patterns of 21-nt *PHAS* loci in strawberry embryos and endosperm. **A** Cluster analysis and heatmap of the expression patterns of all 21-nt *PHAS* loci in embryos and endosperm. **B** The distribution of sRNA reads in the sRNA profiles from the embryo (left) and endosperm (right) tissues at the *PPR* locus. Two tracks showing the abundance (RP10M) of sRNAs in both strands of the *PPR* locus (upper); sRNA sizes are denoted by different colours. The lower track shows the phasing score of each read in this locus. **C** sRNA abundance and phasing score at the *TAR2* locus. The patterns of another *TAR2* locus are similar to those of this *TAR2* locus, as indicated. **D** sRNA abundance and phasing score at the *CM3* locus. The patterns of other *CM3* loci are similar to this *CM3* locus, as indicated

We also examined the expression of each *PHAS* locus (Fig. 6 and Figure S3). Among 81 of the 21-nt *PHAS* loci, 56 showed differential expression in the embryo and endosperm, where 25 and 31 loci accumulated at higher levels in the embryo and endosperm, respectively (Fig. 6A). In this study, we found four *PPR* loci in strawberry, and only one *PPR* locus was specifically expressed in embryos (Fig. 6B). Conversely, other conserved *PHAS* loci, including two *TAR2* and one *AFB2,* showed higher expression in the endosperm (Fig. 6C). In addition, among six *CM3* loci, strawberry also had a higher level in the endosperm (Fig. 6D). However, we observed that most of the 24-nt *PHAS* loci showed embryo-specific expression, and only nine had higher expression in the endosperm, indicating that 24-nt phasiRNAs play important roles in embryo growth and development (Figure S3A). To understand the specific expression patterns of 24-nt *PHAS* loci, sRNA abundance and phasing score at an endosperm-specific zinc transporter protein locus and embryo-specific *MYB* locus were examined, as shown in Figure S3B and C, respectively.

### Identification of miRNA triggers for *PHAS* loci

**Fig. 7:**
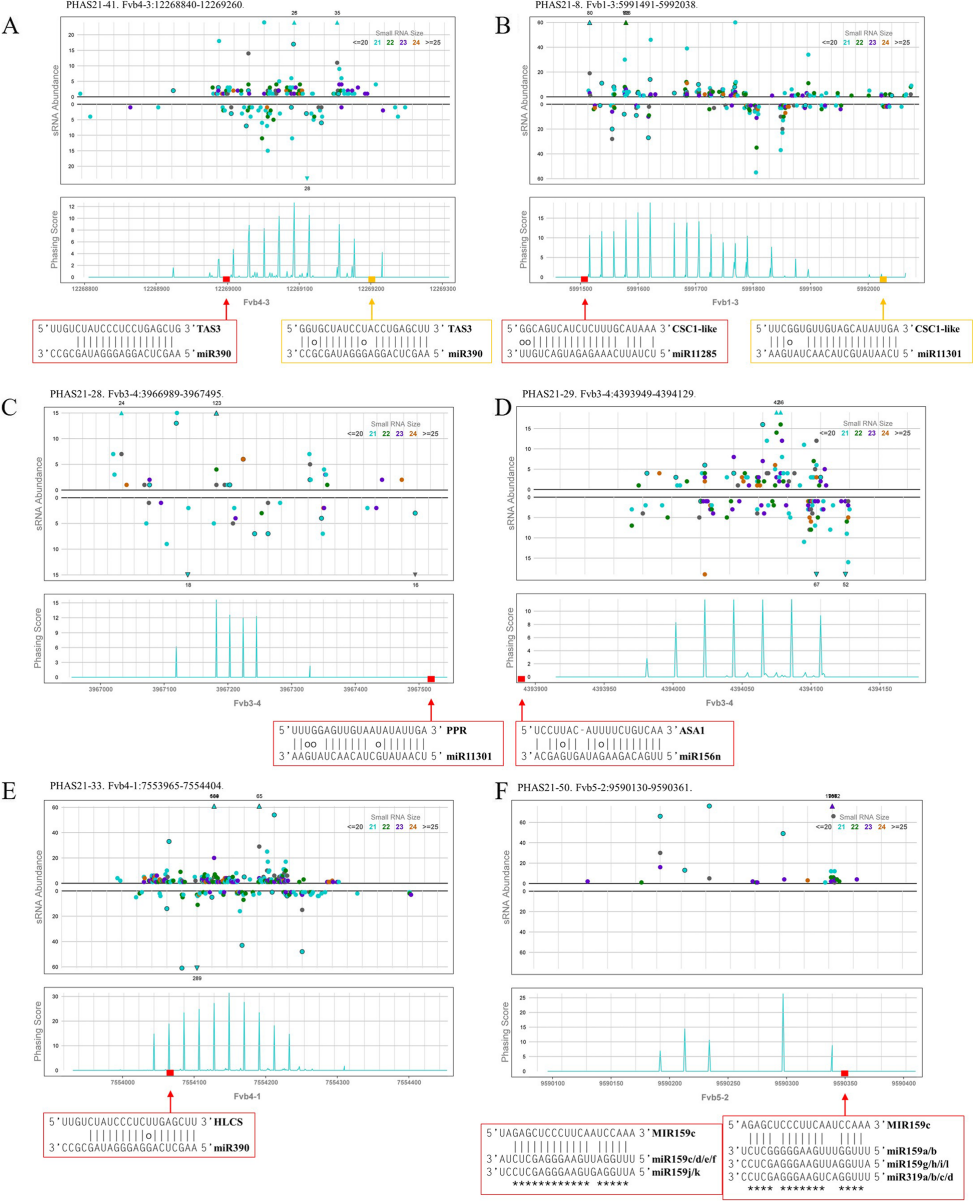
miRNA-triggered 21-nt phasiRNAs in strawberry. **A** sRNA abundance and phasing score are viewed at putative *TAS3* genes and are targeted by miR390 via a “two-hit”-like model in strawberry. **B** Novel *PHAS* locus *CSC1*-like targeted by miR11285 and miR11301. **C** Conserved *PHAS* locus *PPR* was identified in Fvb3-4 and targeted by miR11301. **D** miR156n-triggered phasiRNAs produced from the *ASA1* locus. **E** miR390 also triggered phasiRNA production from the novel *PHAS* locus *HLCS*. **F** Novel *PHAS* locus *MIR159c* targeted by miR159 and miR390

Seventeen families of miRNAs, including 14 known miRNAs and 3 novel miRNAs, were found to trigger phasiRNA production from 21-nt *PHAS* loci. Among the 40 *PHAS* loci triggered by miRNAs, 22 overlapped with annotated genes in the strawberry genome, such as E3 ubiquitin-protein ligase, *PPR*, anthranilate synthase alpha subunit 1 (*ASA1*), and *CM3*. Among the miRNA triggers, most miRNA triggers were 21 nt long, including conserved miRNAs, such as miR390, miR319, miR395 and miR482, and novel miRNAs, such as miRN3 and miRN14, and the remaining miRNA triggers were 20 and 22 nt long. All miRNA trigger-*PHAS* pairs are shown in Table 1. Some conserved miRNA trigger-*PHAS* pairs were found in strawberry, such as miR390-*TAS3* (Fig. 7A) and miR482/2118-ncRNA. In addition, we also found some novel miRNA trigger-*PHAS* pairs, such as miR11301/miR11285-*PPR*/*CSC1*-like (Fig. 7B, C), miR156-*ASA1* (Fig. 7D), miR390-*HLCS* (Fig. 7E), miR319/miR159-*MIR159* (Fig. 7F), miRN11-ncRNA/40S ribosomal protein S29 and miRN14-ncRNA (Table 1).

**Table 1:** Triggers of *PHAS* loci in strawberry

PHAS ID	Putative function	Overlapping gene ID (annotation)	miRNA trigger	miRNA length
PHAS21-4	CSC1-like protein	maker-Fvb1-2-snap-gene-74.69	miR11301a/b/c; miR11285a/b	21;22
PHAS21-8	CSC1-like protein	maker-Fvb1-3-snap-gene-60.54	miR11301a/b/c; miR11285a/b	21;22
PHAS21-9	PPR	Intergenic	miR11285a/b	22
PHAS21-10	ncRNA	Intergenic	miR482a/b/y	22/22/21
PHAS21-13	NULL	maker-Fvb2-1-augustus-gene-234.53 (E3 ubiquitin-protein ligase RNF126-like)	miR482n/s/x; miR2118b	22
PHAS21-17	ncRNA	maker-Fvb3-1-augustus-gene-207.32	miRN14	21
PHAS21-18	ncRNA	maker-Fvb3-1-augustus-gene-207.33	miRN14	21
PHAS21-23	PPR	augustus_masked-Fvb3-2-processed-gene-267.11 (PPR); augustus_masked-Fvb3-2-processed-gene-267.12 (PPR)	miR11301a/b/c; miR11285a/b; miR171d/e/f	21; 22
PHAS21-25	uncharacterized mRNA	Intergenic	miR482a/b	22
PHAS21-26	ncRNA	Intergenic	miRN14	21
PHAS21-27	ncRNA	Intergenic	miRN14	21
PHAS21-28	PPR	snap_masked-Fvb3-4-processed-gene-39.28 (PPR); maker-Fvb3-4-snap-gene-39.55 (PPR)	miR11301a/b/c	21
PHAS21-29	ASA1	augustus_masked-Fvb3-4-processed-gene-44.0 (ASA1)	miR156n	20
PHAS21-30	ncRNA	maker-Fvb3-4-augustus-gene-194.36	miRN14	21
PHAS21-33	HLCS	maker-Fvb4-1-snap-gene-75.30	miR390a/b/c/d/e/f/g/h/i	21
PHAS21-34	NULL	Intergenic	miRN11a/b/c	22
PHAS21-36	ncRNA	Intergenic	miR11301a/b/c; miR164c	21; 20
PHAS21-39	NULL	Intergenic	miR11286a; miRN11a/b/c	21; 22
PHAS21-41	TAS3-like	maker-Fvb4-3-augustus-gene-122.25	miR390a/b/c/d/e/f/g/h/i	21
PHAS21-42	NULL	snap_masked-Fvb4-3-processed-gene-219.12	miR390a/b/c/d/e/f/g/h/i	21
PHAS21-43	NULL	Intergenic	miR390a/b/c/d/e/f/g/h/i	21
PHAS21-44	NULL	Intergenic	miR11285a/b	22
PHAS21-47	TAS3-like	maker-Fvb4-4-augustus-gene-90.46	miR390a/b/c/d/e/f/g/h/i	21
PHAS21-48	NULL	maker-Fvb4-4-snap-gene-187.25 (E3 ubiquitin-protein ligase XBAT32)	miR390a/b/c/d/e/f/g/h/i	21
PHAS21-50	MIR159c	Intergenic	miR319a/b/c/d/e/f/g/h; miR159a/b/c/d/e/f/g/h/i/j/k/l	20; 21
PHAS21-54	MIR159c	Intergenic	miR319a/b/c/d/e/f/g/h; miR159a/b/c/d/e/f/g/h/i/j/k/l	20; 21
PHAS21-56	MIR159c	Intergenic	miR319a/b/c/d/e/f/g/h; miR159a/b/c/d/e/f/g/h/i/j/k/l	20; 21
PHAS21-57	NULL	maker-Fvb6-1-augustus-gene-4.51	miR482p/t	21
PHAS21-60	ncRNA	augustus_masked-Fvb6-1-processed-gene-36.17 (CM3)	miR482p/t	21
PHAS21-62	NULL	Intergenic	miR390a/b/c/d/e/f/g/h/i; miR482c/f/h/u/v/w	21
PHAS21-63	NULL	Intergenic	miR395a/b/c/d/e/f/g/h/i/j/k/l/m/n/o/p; miRN3	21
PHAS21-65	ncRNA	snap_masked-Fvb6-1-processed-gene-319.21	miR482n/s/x; miR2118b	22
PHAS21-66	ncRNA	Intergenic	miRN11a/b/c; miR482d/e/g	22; 21
PHAS21-67	NULL	Intergenic	miR395a/b/c/d/e/f/g/h/i/j/k/l/m/n/o/p; miRN3	21
PHAS21-68	NULL	maker-Fvb6-2-snap-gene-237.55	miR482c/d/e/f/g/h/u/v/w/x; miR2118b	21; 22
PHAS21-69	NULL	augustus_masked-Fvb6-2-processed-gene-304.5 (CM3)	miR482p/t	21
PHAS21-73	NULL	maker-Fvb6-3-augustus-gene-30.84	miR482p/t	21
PHAS21-74	NULL	Intergenic	miR482c/f/h/u/v/w; miR390a/b/c/d/e/f/g/h/i	21
PHAS21-76	uncharacterized mRNA	maker-Fvb6-3-snap-gene-381.45	miR11287a/b; miR482n/s/x; miR2118b; miRN14; miR7125a/b	21; 22
PHAS21-81	40S ribosomal protein S29	snap_masked-Fvb7-4-processed-gene-5.26	miRN11a/b/c	22
PHAS24-2	ncRNA	Intergenic	miRN29	21
PHAS24-11	CSC1-like protein	Intergenic	miR11285a/b	22
PHAS24-14	NULL	Intergenic	miR482d/e/g	21
PHAS24-19	uncharacterized mRNA	Intergenic	miR172a	20
PHAS24-26	uncharacterized mRNA	Intergenic	miR172a	20
PHAS24-29	ncRNA	Intergenic	miRN14	21
PHAS24-30	ncRNA	Intergenic	miRN14	21
PHAS24-42	uncharacterized mRNA	Intergenic	miR11288b/e/i/l/n; miR395a/b/c/d/e/f/g/h/i/j/k/l/m/n/o/p	21
PHAS24-44	uncharacterized mRNA	Intergenic	miR6275a/b/c/d	21
PHAS24-47	ncRNA	maker-Fvb4-3-snap-gene-7.49 (RNA helicase)	miR156r/w; miRN16	20; 22
PHAS24-71	uncharacterized mRNA	Intergenic	miR172a	20
PHAS24-74	ncRNA	Intergenic	miRN29	21

Twelve of the 24-nt *PHAS* loci were triggered by miRNA, including 7 known miRNAs and 3 novel miRNAs. Among the miRNA triggers, most miRNA triggers are 21 nt long, such as miRNA395, miRNA482, miRNA6275, and miRNA11288 and the novel miRNAs miRN14 and miRN29. The remaining miRNA triggers were 20 and 22 nt long, including miR156, miR172, miR11285 and miRN16, respectively. Table 1 also shows miRNA trigger-24-nt *PHAS* pairs, such as miRN29-ncRNA (Figure S4A), miR11285-*CSC1*-like (Figure S4B), miRN14-ncRNA (Figure S4C) and miR11288/miR395-uncharacterized mRNA (Figure S4D).

## Discussion

Bidirectional dialogue between the endosperm and embryo is important for fruit set and development [10, 12–15, 48]. However, studies that focused on the sRNAs involved in endosperm and embryo development have not been found for *Fragaria*.

### Abundance of sRNA in the developing embryo and endosperm

In this study, we constructed 12 sRNA libraries from reciprocally crossed embryo and endosperm tissues from strawberry and sequenced the libraries using high-throughput sequencing technology to gain insight into the identified miRNAs and *PHAS* loci that might be involved in strawberry embryo and endosperm development. Here, we found that the majority of the sRNAs ranged from 20 ~ 25 nt in length, with the 24-nt class being the most abundant in the embryo and endosperm (Fig. 1). The result was similar to previous reports from castor bean [36] and maize [49, 50], while in the green fruit stage and red fruit stage of cultivated strawberry, 21-nt sRNAs represented the most abundant sRNAs [51], suggesting that 24-nt sRNA biogenesis is conserved in embryo and endosperm development.

### Features of the miRNAs in the developing embryo and endosperm

miRNAs are the most functionally important and most widely studied class of sRNA in plants. Our current study confirmed that 352 known miRNAs belonging to 46 miRNA families were correlated with the embryo and endosperm development processes in strawberry. We also found 52 novel miRNAs that have not been reported elsewhere. Among the miRNA families, the miR156 family was the largest family, with 28 members, followed by miR482 and miR171, with 25 and 24 members, respectively (Fig. 2A). These results were similar to those of previous studies, albeit with some differences. For example, Huang et al. [49] found that the miR166 and miR167 families were the largest families, with 15 members in maize endosperm, followed by the miR156 (13 members) and miR171 (11 members) families. The differences in these miRNA family members in different plants revealed that they could function in common or unique regulatory pathways. Compared to the miRNA target genes identified in other plants, many conserved miRNAs, such as miR156, miR160, miR166, miR167, miR395, miR397, and miR408, etc., showed similar targets as their homologues in strawberry [16, 37]. These conserved miRNAs and their targets usually play fundamental regulatory role in plant growth and development. For instance, the conserved miR156 family is involved in regulation of flowering and the vegetative-to-reproductive transition, by cleaving *SPL* transcripts and promoting their translational repression [52]. Moreover, miR156 and miR172 are positively regulated by the transcription factors they target, participates in the stability of juvenile-to-adult phase transition in plants [53, 54]. In plants, miR165 and miR166 share the same function of targeting and cleaving *HD-ZIP* III genes, which act against meristem maintenance [55]. miR397 regulated fruit cell lignification in pear by inhibiting expression of laccase genes which is useful for improving pear fruit quality [56]. However, some conserved miRNAs and most novel miRNAs exhibited species-specific targets in cultivated strawberry. For example, *ARF* transcription factors, which are essential in the early auxin response, were targeted by the miR160 and miR167 families, which is consistent with our results [16, 57]. Our study showed that miR160 family members were all more highly expressed in the embryo than in the endosperm and targeted *ARF17* and *ARF18*. In the miR167 family, many members were highly expressed in the embryo, while miR167j, miR167k and miR167l were highly expressed in the endosperm. Their target genes were *ARF6* and *ARF8*. Moreover, we found that *ARFs* were also targeted by miR159. The results of our study showed that miR159 family members, including miR159m, miR159n, miR159o, miR159p, miR159q and miR159r, showed endosperm-specific expression (Figure S5) and targeted *ARF23* (Table S3). Previous research showed that *ARF23* is a pseudogene in *A. thaliana*, and its biological function and ability to bind DNA is not known [58–61]. Among the various functional mechanisms of pseudogenes, antisense transcription-related regulation and competition with miRNA and RNA-binding proteins appear to be common in humans [62, 63]. The genetic functions and evolutionary trends for *ARF23* in the strawberry genome are unknown. Additionally, it is unclear how the miR159 family that was predicted to target *ARF23* regulates the development of strawberry endosperm and embryos. These are interesting issues that require further investigation.

In addition, we classified the miRNA families according to their expression level profiles to explore diverse molecular and physiological pathways of miRNAs in strawberry embryos and endosperm. We found that some of the conserved miRNA families, such as miR164, miR167, miR168 and miR395, showed conserved expression in the developing reciprocal cross endosperm, and other conserved miRNA families, including miR159, miR160, miR162, miR166, miR171, miR319, miR395, miR399, and miR535, showed conserved expression in the developing reciprocal cross embryo (Fig. 3A). This result is similar to the research on embryos and endosperm in maize [64].

### PhasiRNAs involved in the developing embryo and endosperm

In addition to miRNAs, phasiRNAs have been shown to be involved in developmental regulation and stress responses in flowering plants. Genome-wide identification of phasiRNAs is an important tool that can be used to investigate gene regulation involving small RNAs in legumes [24], soybean [65], tomato [66], litchi [26], *Panax notoginseng* [67], wheat [68], and pummelo [69]. In the woodland strawberry *F. vesca*, a total of 515 *PHAS* loci, including 192 21-nt *PHAS* loci and 323 24-nt *PHAS* loci, were identified [27]. In our study, 81 21-nt *PHAS* loci and 75 24-nt *PHAS* loci were identified in the cultivated strawberry *F.* × *ananassa* genome (Table S4). However, only 49.38% (40/81) of the 21-nt *PHAS* loci and 16% (12/75) of the 24-nt *PHAS* loci were identified as targets of miRNA triggers. Many studies have revealed that a few phasiRNA pathways are highly conserved alongside conserved miRNAs in land plants [50]. *TAS3* is arguably the most well-conserved *PHAS* locus, as it has been identified across a broad range of species, and the miR390-*TAS3*-*ARF* pathway is the most conserved and archetypal [44, 70]. Xia et al. [38] identified two *TAS3* genes, named *TAS3L* and *TAS3S*, in *F. vesca*. Cultivated strawberry also has two *TAS3* genes targeted by miR390, but we did not find other *TAS* genes in the embryo and endosperm, which suggested that *TAS3* is primarily conserved in *Fragaria*. A number of *PPR*s were shown to generate phasiRNAs and to be conserved, especially in eudicots. The pathway for the generation of *PPR* phasiRNAs includes a small number of diverse trigger miRNAs. For instance, miR161, miR400, and miR173 in *Arabidopsis* [20, 71, 72], miR1509 in legumes [24], miR7122 in Rosaceae [73], and miR11285 in *F. vesca* [38]. In this study, our research showed that *PPR* generated phasiRNAs by triggering miR11285 or miR11301 (Fig. 7C and Table 1); this result was similar to that in *F. vesca*. We also identified some conserved *PHAS* loci in strawberry, i.e., *TAR2*, *NAC*, and *AFB2* (Fig. 5A). As an auxin biosynthetic gene, *TAR2* plays a role in the indole-3-pyruvic acid (IPA)-dependent route for the conversion of Trp to IAA, which is the main mechanism for the de novo synthesis of auxin in plants and is required for proper embryo development, lateral root growth and several developmental processes in *Arabidopsis* [74–78]. As previously described in *Arabidopsis*, *FaTAR2* works as a Trp aminotransferase in strawberry and is involved in auxin biosynthesis. Transient silencing of this gene was accompanied by a diminished responsiveness to auxin in ripening receptacles [48]. *NAC*s are a family of genes specific to plants that play a role in a diverse set of developmental processes; their functions are partially similar to that of *TAR2*. For example, *NAC1* is also involved in the auxin-signalling pathway to promote lateral root development, and in Arabidopsis, miR164-directed regulation of the NAC-domain gene *CUC1* is necessary for normal embryonic development [79, 80]. In addition, *NAC1*, as a transcriptional activator, regulates the expression of two auxin-responsive genes, *AIR3* and *DBP* [79]. *AFB2* has positive regulatory roles as an auxin receptor in the regulation of fruit development compared with the *AFB* gene family [81, 82] and has the highest expression level in the early fruit development stage in cucumber and the diploid strawberry *F. vesca* [10, 83]. Moreover, *AFB2* plays a major role in the control of jasmonic acid conjugation through the *ARF6*/*ARF8* auxin signalling module during Arabidopsis adventitious rooting initiation [84]. In addition, the *PHAS* genes *ASA1* and *CM3* were newly identified in strawberry and found to play an important role in the shikimate pathway [46]. The shikimate pathway serves as an important precursor for auxin biosynthesis, and Trp accumulation occurs via this pathway to sustain auxin production [76]. Overall, we suggest that the biosynthesis of auxin-related phasiRNAs is an indispensable regulator of early endosperm and embryo development and of receptacle fruit set in strawberry.

## Conclusions

In this study, a total of 404 miRNAs (352 known and 52 novel) could be found in strawberry embryo and endosperm tissues, of which four and nine conserved miRNA families displayed conserved expression in the endosperm and embryo, respectively. We also identified 81 *PHAS* loci producing 21-nt phasiRNAs and 75 *PHAS* loci producing 24-nt phasiRNAs. Among them, some auxin signal-related genes, *CM3*, *TAR2*, *AFB2*, *ASA1*, *NAC* and *TAS3,* were found, which demonstrates that *PHAS* locus-mediated IAA biosynthesis is important for endosperm and embryo during early fruit growth. Additionally, some auxin signal-related conserved (miR390-*TAS3*) and novel (miR156-*ASA1*) trigger-*PHAS* pairs were identified. The above results improve our knowledge of sRNAs in strawberry embryo and endosperm development and provide candidate *PHAS* loci for the early developmental stage of fruit in *F.* × *ananassa*.

## Materials and methods

### Plant materials and growth conditions

Two cultivated strawberry cultivars, *F.* × *ananassa* cultivars ‘Benihoppe’ and ‘Sweet Charlie’, were used as materials for sRNA identification and were grown in the Baima Teaching and Scientific Research Base of Nanjing Agricultural University, Nanjing, China. Developing fruits (receptacles with achenes), BS achenes (‘Benihoppe’ as the female, pollinated with ‘Sweet Charlie’) and SB achenes (‘Sweet Charlie’ as the female, pollinated with ‘Benihoppe’) from reciprocal crosses between ‘Benihoppe’ and ‘Sweet Charlie’ were collected at 12 DAP. The embryo and endosperm of a developing ovary (i.e., achene) were hand-dissected under a stereomicroscope, washed with tissue separation buffer [85], and then frozen immediately in liquid nitrogen. Approximately 2500 embryos and endosperm samples were dissected and pooled into one biological replicate, and three biological replicates were collected.

### Small RNA library construction and sequencing

After total RNA of embryos and endosperm from each reciprocal cross achene was extracted by a TRIzol reagent kit (Invitrogen, Carlsbad, CA, USA), the small RNAs in a size range of 16–30 nt were enriched by 15% denaturing polyacrylamide gel electrophoresis (PAGE). Then, 3’ adapters were added, and the 36–44 nt RNAs were enriched. Next, 5’ adapters were ligated to the RNAs. The ligation products were reverse transcribed by PCR amplification, and the 140–160 bp PCR products were enriched to generate a cDNA library and sequenced using Illumina HiSeq™ 2500.

### Small RNA sequencing analysis

Clean tags were obtained by removing the adaptor sequences, filtering out the low-quality tags and eliminating short lengths (< 14 nt). Then, all the clean reads were aligned with small RNA sequences in GenBank databases [32] and the Rfam 11.0 database [33] to identify and filter the ribosomal RNA (rRNA), transfer RNA (tRNA), small cytoplasmic RNA (scRNA), small nuclear RNA (snRNA) and small nucleolar RNA (snoRNA). After removing noncoding RNAs, the clean small RNA sequences were mapped to the *F.* × *ananassa* ‘Camarosa’ genome [34] to obtain unique reads with abundance and location information based on the genome using the SOAP 2.0 program (http://soap.genomics.org.cn/). The unique RNA sequences that perfectly matched the genome were subjected to subsequent analysis. Sequence reads that mapped to exons and introns of mRNA were removed to avoid mRNA degradation products, and the tags that mapped to repeat sequences were also removed.

### Identification of known and novel miRNAs and their targets

To identify the known miRNAs, the unique RNA sequences that perfectly matched the *F.* × *ananassa* ‘Camarosa’ genome [34] were searched against the mature or precursor miRNAs of cultivated strawberry and mature plant miRNA sequences in the miRBase 22 database [35] by using the sRNAanno database [43]. The small RNA sequences that perfectly matched known miRNAs were considered to be known miRNAs. Potential miRNA precursor sequences were obtained by aligning the reads to the strawberry reference genome, and their secondary structure were predicted by using the RNAfold [86]. The results of secondary structure were analysed to verify characteristic miRNAs based on the previous criteria [28]. Sequences that did not find matches in the miRBase were identified as novel miRNAs by sRNAanno [43], which uses a set of well-established criteria, as previously reported [87]. Secondary structures of miRNAs are shown by RNAfold [86].

Based on the sequences of the known miRNAs and novel miRNAs, the software PatMatch (Version 1.2) [88] was used to predict target genes. The following criteria were applied: (1) no more than four mismatches between the sRNA and the target; (2) no more than two adjacent mismatches in the miRNA/target duplex; and (3) no mismatches in positions 10–11 of the miRNA/target duplex. (4) The minimum free energy (MFE) of the miRNA/target duplex should be ≥ 74% of the MFE of the miRNA bound to its perfect complement.

### Identification of differentially expressed miRNAs in the embryo and endosperm of strawberry

To screen differentially expressed miRNAs during embryo and endosperm development in strawberry, the expression level of miRNAs in each sample was calculated and normalized to transcripts per million (TPM) using miRNA counts. The significant differentially expressed miRNAs were determined by a fold change ≥ 2 and *P* value < 0.05 for each comparison. The heatmap of miRNA expression was generated using TBtools [47].

### qRT‒PCR analysis of differentially expressed miRNAs and their target genes

For quantification of mature miRNAs, we used the tailing-reaction qRT‒PCR method. Tailing-reaction RT‒PCR was performed using a miRNA 1st strand cDNA synthesis kit (Accurate Biotechnology, China) with general RT tailing-reaction primers. TB Green® Premix Ex Taq™ (TaKaRa, Japan) and tailing-reaction 3’ primer (Accurate Biotechnology, China) were used for qPCR on a QuantStudio 5 system (ABI, USA). For expression analysis of target genes, qRT‒PCR was performed using a QuantStudio 5 system (ABI, USA), with a PrimeScript™ RT reagent Kit (TaKaRa, Japan) for reverse transcription and TB Green® Premix Ex Taq™ (TaKaRa, Japan) for qPCR. Three independent biological replicates were used, and 26S rRNA and *EF-1α* were used as the reference genes for the analysis of the expression of miRNAs and target genes, respectively. The relative expression levels of miRNAs and target genes were calculated by using the 2^−ΔΔCt^ method. All primers used in this experiment are listed in Table S5.

### Identification and analyses of putative *PHAS* loci and miRNA triggers

To investigate phasiRNA biogenesis and regulation in embryos and endosperm, a genome-wide search for *PHAS* loci was conducted based on small RNA reads by using the sRNAanno database [43]. Loci with a *P* value < 0.001 were considered to be reliable *PHAS* loci. These *PHAS* loci were refined for putative annotation by aligning their sequences to the NCBI Nucleotide Collection (nr/nt) database (https://www.ncbi.nlm.nih.gov/) and the PlantRep database [89]. A potential miRNA trigger of *PHAS* loci was also predicted using the sRNAanno database [43].

## Supplementary Information

**Additional file 1:** **Figure S1.** Statistical analysis of sequencing reads in twelve libraries in strawberry reciprocally crossed embryo and endosperm. BS and SB represent ‘Benihoppe’ (♀) × ‘Sweet Charlie’ (♂) and ‘Sweet Charlie’ (♀) × ‘Benihoppe’ (♂), respectively. em: embryo; en: endosperm; rRNA: ribosomal RNA; tRNA: transfer RNA; snoRNA: small nucleolar RNA; snRNA: small nuclear RNA. For each tissue, three biological replicates (em1, em2, and em3; en1, en2, and en3) were performed.

**Additional file 2:** **Figure S2.** The abundant of miRNA families in strawberry reciprocally crossed embryo and endosperm.

**Additional file 3: Figure S3.** Expression patterns of 24nt *PHAS* loci in strawberry embryos and endosperm. A. Heatmap of all 24nt *PHAS* loci expression patterns in embryos and endosperm. B. sRNA abundance and phasing score at the zinc transporter protein locus. C. sRNA abundance and phasing score at the *MYB* locus.

**Additional file 4: Figure S4.** The miRNA-triggered 24nt phasiRNAs in strawberry. A. sRNA abundance and phasing score are viewed at ncRNA is targeted by miRN29. B. Novel *PHAS* locus *CSC1*-*like* targeted by miR11285. C. ncRNA targeted by miRN14. D. The novel *PHAS* locus targeted by miR395 and miR11288.

**Additional file 5: Figure S5.** Some miR159 family members was predicted targeted *ARF23* that showed an endosperm-specific expression in strawberry.

**Additional file 6: Table S1.** Distribution of the sRNA sequencing in the 12 libraries.

**Additional file 7: Table S2.** Known and novel miRNAs in the developing embryo and endosperm.

**Additional file 8: Table S3.** Prediction and analysis miRNA target genes.

**Additional file 9: Table S4.** The basic information and annotation of the predicted *PHAS* loci in the strawberry.

**Additional file 10: Table S5.** The primers for qRT-PCR.

## Data Availability

The raw sRNA sequencing data have been submitted to the NCBI SRA database under the accession numbers SAMN28078473, SAMN28078474, SAMN28078475 and SAMN28078476.

## Abbreviations

ABA: Abscisic acid
AFB2: Auxin signalling F-Box 2
ARF: AUXIN RESPONSIVE FACTOR
ASA1: Anthranilate synthase alpha subunit 1
BS: ‘Benihoppe’ as the female, pollinated with ‘Sweet Charlie’
CM3: Chorismate mutase 3
DAP: Days after pollination
EDM2: ENHANCED DOWNY MILDEW 2
FT1: Flowering locus T1
hc-siRNAs: Heterochromatic small interfering RNAs
IPA: Indole-3-pyruvic acid
LEC2: B3 domain-containing transcription factor LEC2
MFE: Minimum free energy
miRNAs: MicroRNAs
MYB: V-myb avian myeloblastosis viral oncogene homologue
NAC: (NAM, ATAF and CUC) proteins
NB-LRR: Nucleotide-binding leucine-rich repeat receptors
PAGE: Polyacrylamide gel electrophoresis
PHAS loci: PhasiRNA-producing loci
phasiRNAs: Phased small interfering RNAs
PPR: PENTATRICOPEPTIDE REPEAT
qRT‒PCR: Quantitative reverse transcription PCR
RdDM: RNA-directed DNA methylation
RDR6: RNA-DEPENDENT RNA POLYMERASE6
rRNAs: Ribosomal RNAs
SB: ‘Sweet Charlie’ as the female, pollinated with ‘Benihoppe’
scRNAs: Small cytoplasmic RNAs
SEM: Scanning electron microscopy
siRNAs: Short interfering RNAs
snoRNAs: Small nucleolar RNAs
snRNAs: Small nuclear RNAs
sRNAs: Small RNAs
TAR2: Tryptophan aminotransferase-related 2
TPM: Transcripts per million
tRNAs: Transfer RNAs
Trp: Tryptophan
