# Supplementary figures and images for: Identification and characterization of miRNAs and PHAS loci related to the early development of the embryo and endosperm in Fragaria × ananassa
Source: BMC Genomics. 2022 Sep 8;23:638. doi: 10.1186/s12864-022-08864-3 (PMC9454143; doi:10.1186/s12864-022-08864-3)

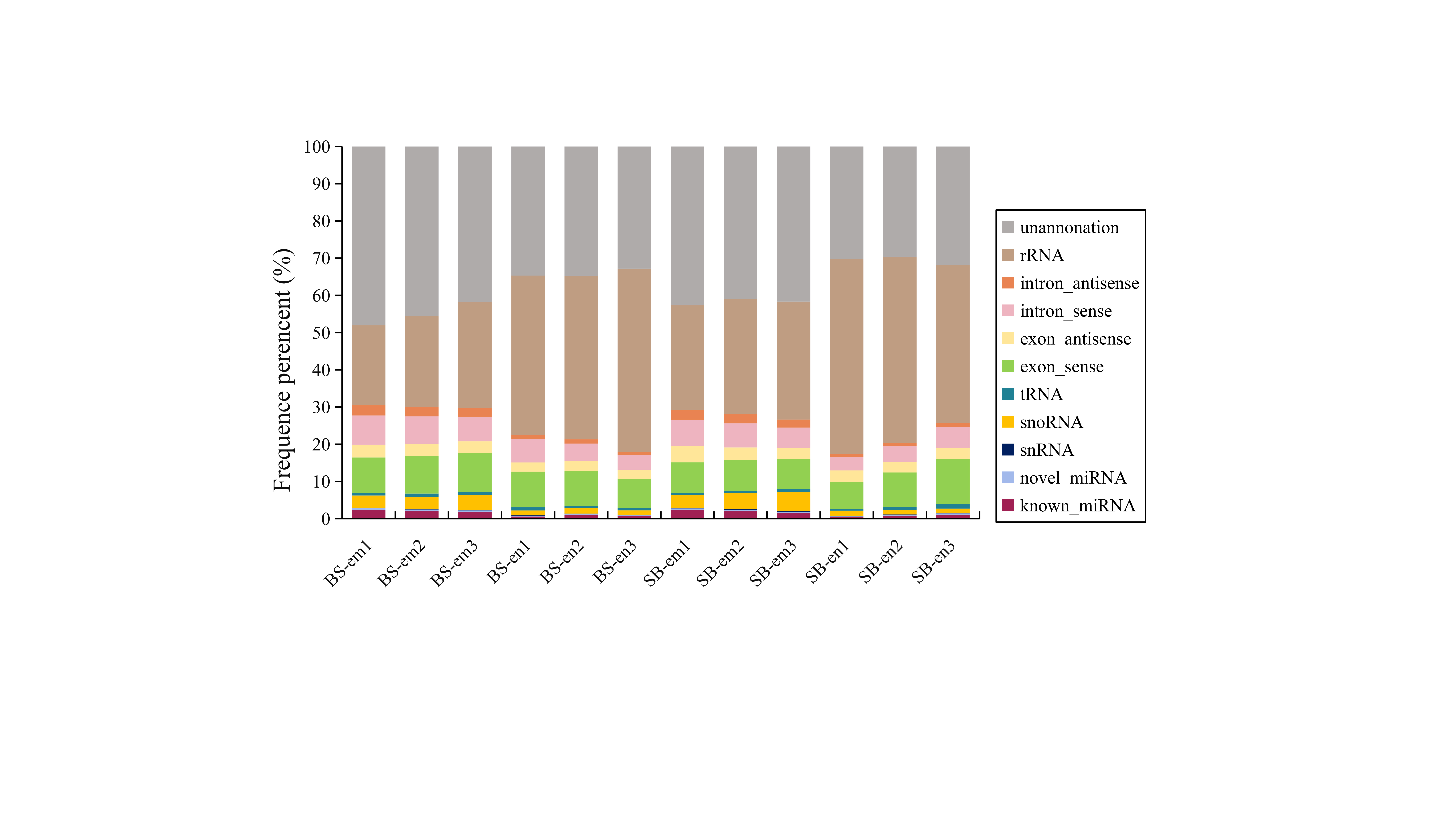

Supplement: Supplementary file 1 — Additional file 1: Figure S1. Statistical analysis of sequencing reads in twelve libraries in strawberry reciprocally crossed embryo and endosperm. BS and SB represent ‘Benihoppe’ (♀) × ‘Sweet Charlie’ (♂) and ‘Sweet Charlie’ (♀) × ‘Benihoppe’ (♂), respectively. em: embryo; en: endosperm; rRNA: ribosomal RNA; tRNA: transfer RNA; snoRNA: small nucleolar RNA; snRNA: small nuclear RNA. For each tissue, three biological replicates (em1, em2, and em3; en1, en2, and en3) were performed. [file 12864_2022_8864_MOESM1_ESM.jpg]

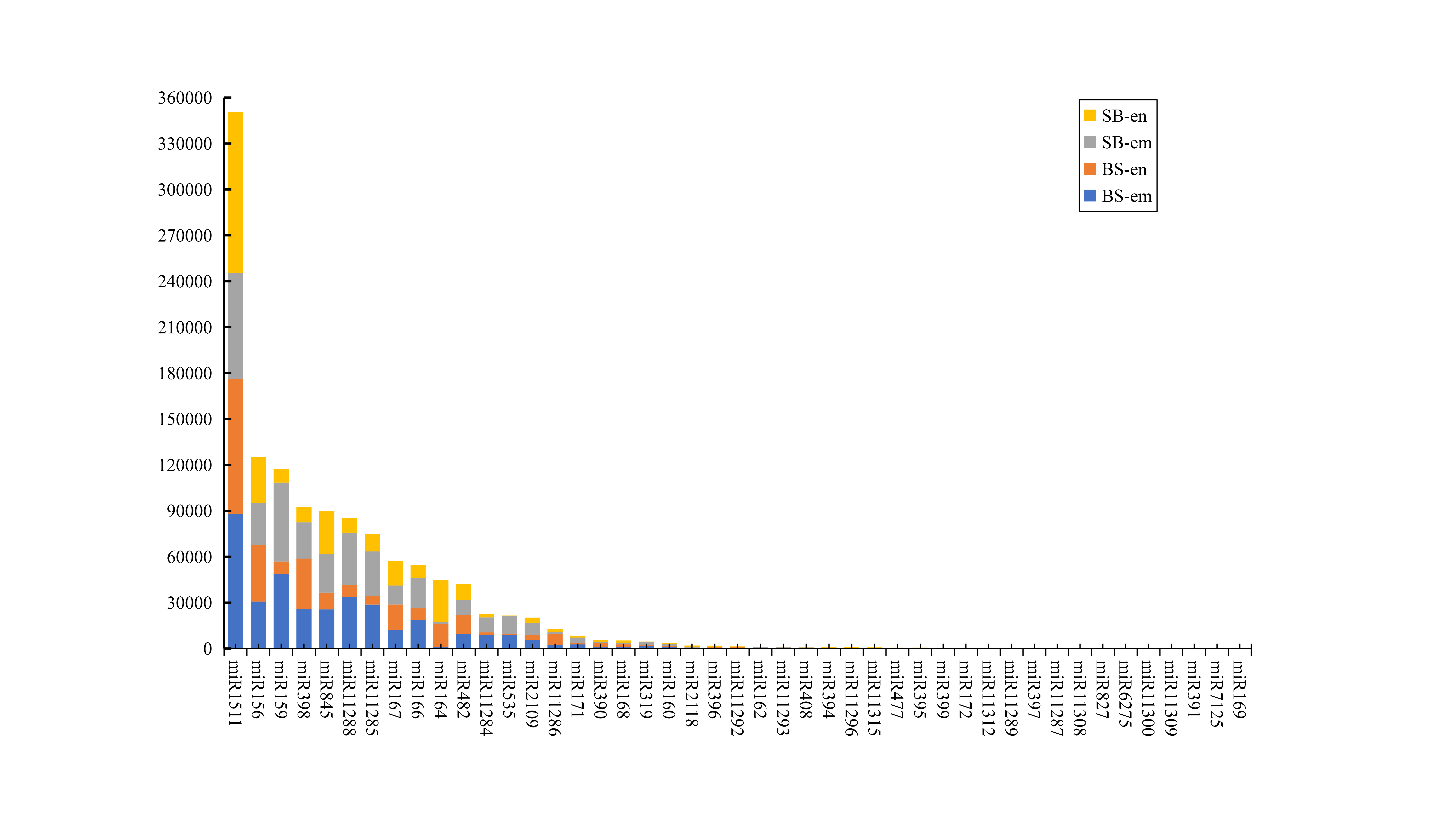

Supplement: Supplementary file 2 — Additional file 2: Figure S2. The abundant of miRNA families in strawberry reciprocally crossed embryo and endosperm. [file 12864_2022_8864_MOESM2_ESM.jpg]

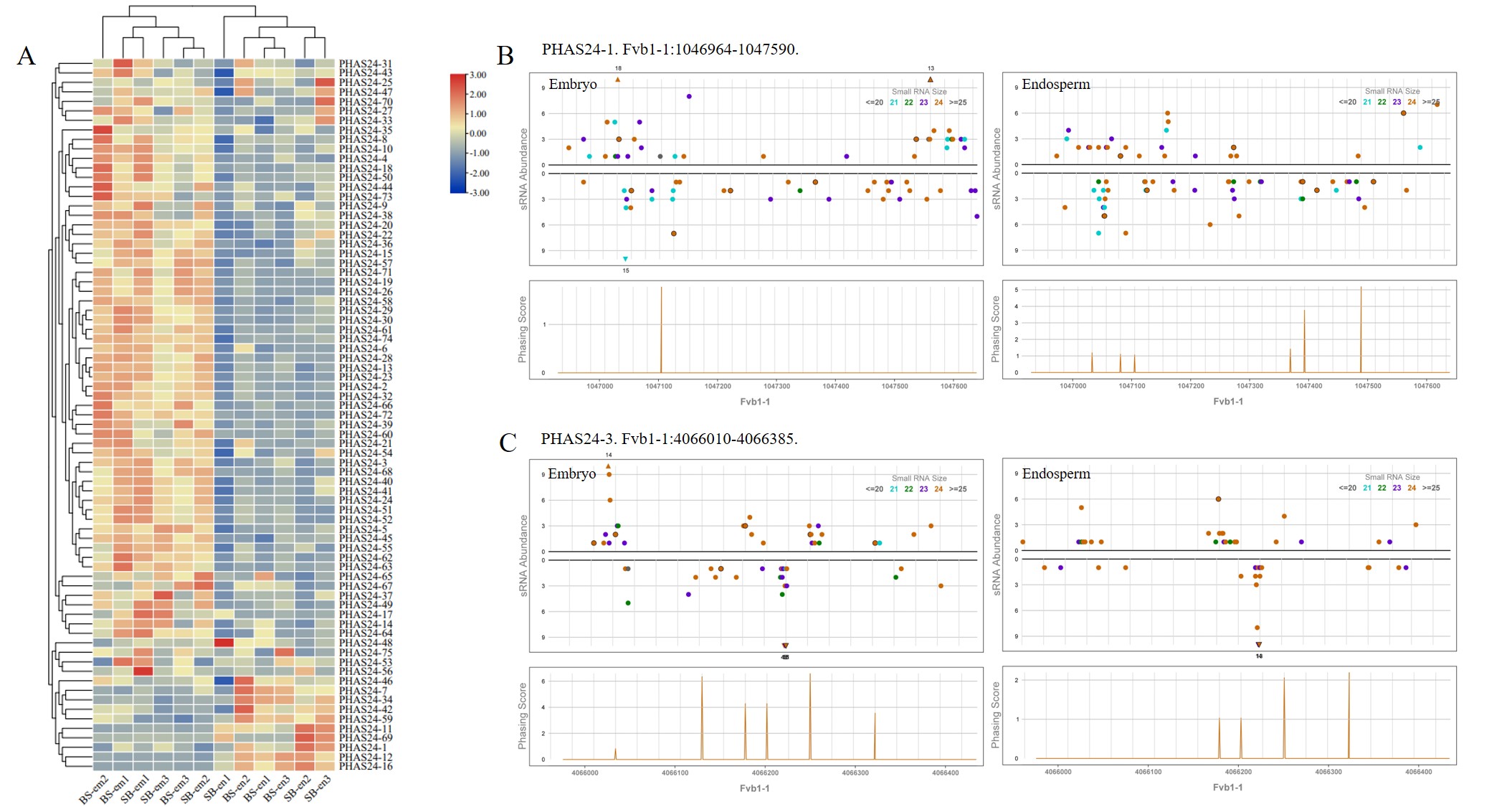

Supplement: Supplementary file 3 — Additional file 3: Figure S3. Expression patterns of 24nt PHAS loci in strawberry embryos and endosperm. A. Heatmap of all 24nt PHAS loci expression patterns in embryos and endosperm. B. sRNA abundance and phasing score at the zinc transporter protein locus. C. sRNA abundance and phasing score at the MYB locus. [file 12864_2022_8864_MOESM3_ESM.jpg]

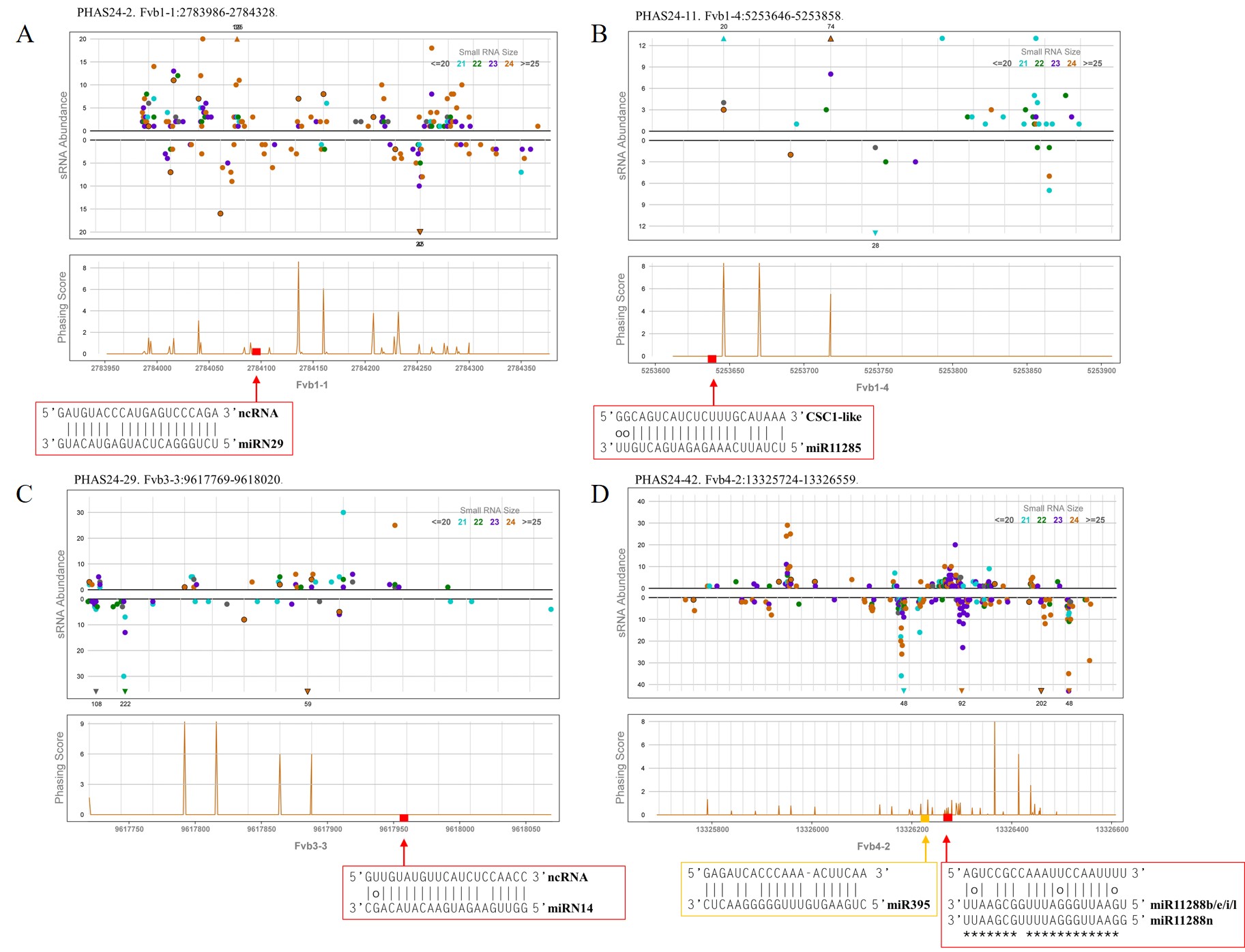

Supplement: Supplementary file 4 — Additional file 4: Figure S4. The miRNA-triggered 24nt phasiRNAs in strawberry. A. sRNA abundance and phasing score are viewed at ncRNA is targeted by miRN29. B. Novel PHAS locus CSC1-like targeted by miR11285. C. ncRNA targeted by miRN14. D. The novel PHAS locus targeted by miR395 and miR11288. [file 12864_2022_8864_MOESM4_ESM.jpg]

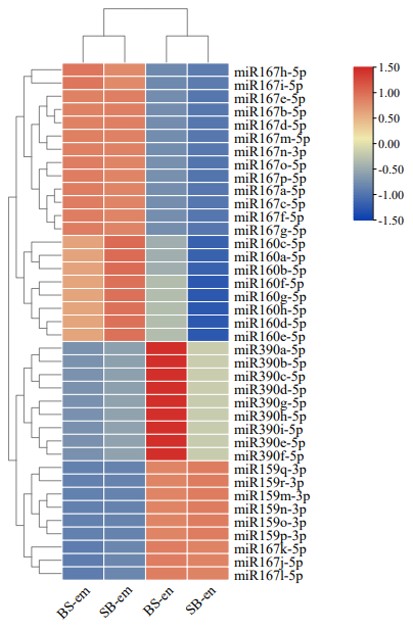

Supplement: Supplementary file 5 — Additional file 5: Figure S5. Some miR159 family members was predicted targeted ARF23 that showed an endosperm-specific expression in strawberry. [file 12864_2022_8864_MOESM5_ESM.jpg]
